# Changes in Anterior Chamber Depth after Phacoemulsification in Pseudoexfoliative Eyes and their Effect on Accuracy of Intraocular Lens Power Calculation

**DOI:** 10.4274/tjo.56659

**Published:** 2016-12-01

**Authors:** Sirel Gür Güngör, Ahmet Akman, Leyla Asena, Mustafa Aksoy, Almila Sarıgül Sezenöz

**Affiliations:** 1 Başkent University Faculty of Medicine, Department of Ophthalmology, Ankara, Turkey

**Keywords:** anterior chamber depth, mean absolute error, phacoemulsification surgery, pseudoexfoliation syndrome

## Abstract

**Objectives:**

To compare anterior chamber depth (ACD) changes after phacoemulsification surgery in patients with pseudoexfoliation syndrome (PEX) and normal patients using an anterior segment imaging method. Another aim of this study was to evaluate the effect of these changes on the accuracy of intraocular lens (IOL) power calculation and postoperative refraction.

**Materials and Methods:**

Twenty-two eyes of 22 patients with PEX and 30 eyes of 30 normal patients who underwent uneventful phacoemulsification surgery and IOL implantation were included in the study. The ACD of all patients was evaluated preoperatively and at 3 months postoperatively with the ALLEGRO Oculyzer (WaveLight® Oculyzer™ II, Alcon, Novartis)-Scheimpflug imaging system.

**Results:**

The postoperative mean ACD values were significantly larger than the preoperative ACD values in both groups (p<0.001 for both groups). The pre- to postoperative change in ACD was 0.46±0.3 mm in the PEX group, which was a larger change than seen in the normal patients (0.12±0.1 mm) (p=0.04). The mean absolute errors (MAE) calculated with different IOL formulas (SRK/T, Haigis, Hoffer and Holladay 1 formulas) were comparable and no statistically significant difference was observed between the two groups (p=0.21).

**Conclusion:**

Phacoemulsification induces more significant ACD changes in patients with PEX compared to normal patients. However, the MAE did not differ significantly between the groups.

## INTRODUCTION

Accurate intraocular lens (IOL) power calculation in cataract surgery is essential to achieve the postoperative target refraction and high patient satisfaction.^1^ The accuracy of IOL power calculation mainly depends on the accuracy of three factors: preoperative biometric data (axial length (AL), anterior chamber depth (ACD), lens thickness, and keratometric index), IOL power calculation formulas, and IOL power quality control by the manufacturer.^1,2,3^ The true effective lens position (ELP) is defined as the effective distance from the anterior surface of the cornea to the lens plane.^4^ ELP is the only parameter that cannot be measured preoperatively. Most biometric formulas estimate ELP mathematically by using keratometric data and AL. ELP plays a key role in the accuracy of IOL power formulas.^5^ Thus, a difference of only 1 mm in IOL position leads to approximately 1.25 diopter (D) change in refraction.^6,7^ Therefore, correct estimation of ELP is a critical step in IOL power prediction.^3^

Patients with pseudoexfoliation syndrome (PEX) frequently undergo phacoemulsification and IOL implantation for cataract surgery; however, according to our clinical observations, refractive outcomes for (PEX) patients are less accurate than the normal population. We thought that this may be due to difficulties in calculating the ELP arising from zonular laxity in (PEX) patients.

The aim of this study was to compare the ACD changes in patients with (PEX) and normal eyes after phacoemulsification. Another aim of this study was to evaluate the effect of these changes on the postoperative refraction.

## MATERIALS AND METHODS

A total of 52 eyes (22 eyes affected by (PEX) and 30 normal eyes) of 52 patients (22 men, 30 women) who underwent uneventful phacoemulsification surgery and IOL implantation performed between May 2013 and May 2014 were enrolled in this prospective study. Patients with corneal pathology, glaucoma, uveitis, previous eye surgery or eye trauma, posterior segment pathology, diabetes, and those using topical or systemic medications that might influence anterior segment parameters were excluded from the study.

In patients undergoing sequential bilateral phacoemulsification cataract surgery, we randomly selected (by coin toss) only one eye to be included in the study. Informed consent was obtained from all patients in compliance with the World Medical Association’s Declaration of Helsinki. The local institutional review board approved the protocol.

One surgeon (A.A.) performed all operations under topical anesthesia. In all eyes, a 2.2 mm clear corneal incision through a temporal approach was created. Through this incision, a continuous curvilinear capsulorhexis measuring approximately 5.5 mm in diameter was performed. The hydrodissection was followed by phacoemulsification of the nucleus and cortex aspiration. The lens capsule was inflated with an ophthalmic viscosurgical device and the same foldable hydrophobic acrylic IOL (SN60WF AcrySof; Alcon Laboratories, Fort Worth, TX, USA) was placed in the capsular bag. The corneal wound was not sutured. There were no intraoperative or postoperative complications for any patients.

**Figure 1:**
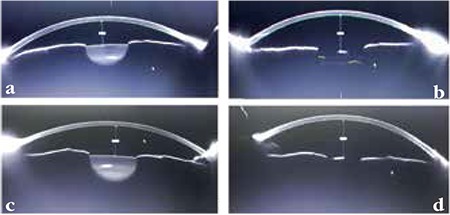
ALLEGRO Oculyzer-Scheimpflug imaging system showing the changes in the anterior chamber depth induced by cataract surgery in an eye with pseudoexfoliation syndrome and in a normal eye. In the eye with pseudoexfoliation syndrome, the anterior chamber depth increased from 2.50 mm (a) to 3.85 mm (b). In the normal eye, the anterior chamber depth increased from 2.90 mm (c) to 3.80 mm (d)

The ACDs of all patients were evaluated preoperatively and at the third month postoperatively with the ALLEGRO Oculyzer (WaveLight® Oculyzer™ II, Alcon, Novartis) - Scheimpflug imaging system, which is a diagnostic device based on the Pentacam HR technology, providing non-contact measurement and analysis of the complete anterior eye segment. The measurements were obtained by two blinded, independent observers (L.A. and M.A.) and averaged for analysis. All measurements were obtained under standard dim light conditions and without pupil dilation with the patient seated using a chinrest and forehead strap. Three measurements were obtained in each study eye and the mean value was used in quantitative analyses. Postoperative ACD was determined using inbuilt calipers on the Scheimpflug image (Figure 1) because of the possible failure to identify the anterior surface of the IOL.^8^

Preoperative AL, keratometric power, and ACD were also measured using the IOL-Master (Zeiss IOL-Master 500, Carl Zeiss Meditec, Jena, Germany). Preoperative biometric data in both groups were used in the IOL power formula to calculate the power of the implanted IOL, which was used to calculate predicted refractive spherical equivalent (SE). The power of the implanted IOL was determined using Haigis, SRK/T, Hoffer, and Holladay 1 formulas. Postoperative refractive errors were measured 3 months after cataract surgery using automatic refracto-keratometry (RKT-7700; Nidek, Hiroshi, Japan). The mean absolute error (MAE) was defined as the average of the absolute value of the differences between the actual and predicted SE of the postoperative refractive error.

### Statistical Analysis

Statistical analysis was performed with SPSS for Windows version 13.0 (SPSS Inc, Chicago, IL, USA). All data were reported as means ± standard deviations (SD). Normality of continuous variables in a group was determined by Shapiro-Wilks test. The variables showed normal distribution (p>0.05). Therefore, a paired t-test, chi-square test and Mann-Whitney U-test were used to compare variables between the pre- and postoperative periods. The predictive accuracy of the formula was analyzed by comparing the MAEs. A paired t-test was used to compare the between-group difference in MAEs calculated by the Haigis, SRK/T, Hoffer, and Holladay 1 formula. A repeated-measures analysis of variance was used to determine the between-group difference. The difference in MAEs between the formulas was assessed using the Tukey multiple comparison test. A value of p<0.05 was considered statistically significant.

## RESULTS

Mean age was 68.3±7.3 years in the (PEX) group (8 men, 14 women) and 67.4±5.8 years in the normal group (14 men, 16 women). Preoperative refractive status was -1.42 D in (PEX) patients and -1.26 D in normal patients. There was no statistically significant difference with respect to gender and age between groups (p>0.05). Patients’ characteristics are listed in Table 1.

Mean IOL power was 21.21±2.1 D (range, 17.5-23.5 D) in the (PEX) group and 21.70±2.2 D (range, 17.5-25 D) in the normal group (p=0.67). The AL measured by the IOL-Master was 23.78±1.37 mm (range, 22.02-25.53 mm) in the (PEX) group and 23.48±0.80 mm (range, 21.79-25.03 mm) in the normal group (p=0.12). There was no statistically significant difference in mean keratometric values between groups (43.37±2.20 D in the (PEX) group; 43.39±1.80 D in the normal group; p=0.23).

The mean preoperative ACD was 3.04±0.5 mm in the (PEX) group and 3.26±0.3 mm in normal patients (p=0.28). At postoperative month 3, the mean ACD was 3.52±0.3 mm in the (PEX) group and 3.38±0.2 mm in normal patients (p=0.35). The postoperative mean ACD values were significantly higher than the preoperative ACD values in both groups (p<0.0001 for both groups.). The difference between postoperative and preoperative ACD values was 0.46±0.3 mm in the (PEX) group, which was a greater change than in the normal patients (0.12±0.1 mm) (p=0.04).

The MAEs calculated by the SRK/T, Haigis, Hoffer and Holladay 1 formulas were comparable between the 2 groups (p>0.05) (Table 2) and no statistically significant difference was observed with different formulas in the same group of patients (p=0.21, Tukey multiple comparison).

## DISCUSSION

Reports in the literature concerning the overall ocular dimensions of eyes with (PEX) are controversial. Earlier studies that looked at ACD in eyes with (PEX) did not detect significant shallowing of the anterior chamber in comparison with normal control eyes.^9,10^ In contrast, one recent study that analyzed age-and gender- matched patients with and without (PEX) found significantly smaller anterior segments in eyes with (PEX).^11^ In addition, the anterior chamber volume was found to be significantly smaller in eyes with (PEX) than in eyes without (PEX).^12^ In a study by Doganay et al.^13^ evaluating anterior segment parameters in patients with (PEX) syndrome or (PEX) glaucoma with the Pentacam-Scheimpflug imaging system, ACD in the (PEX) glaucoma group (2.49±0.39 mm) was found to be significantly lower than the control group and there was no statistically difference between the (PEX) group (2.50±0.29 mm) and the control group (2.60±0.31 mm). In our study, the preoperative ACD values in the (PEX) group (3.04 mm) were lower than the normal group (3.26 mm) but the difference was not statistically significant.

The ALLEGRO Oculyzer is an easy-to-use, non-contact tomography system that uses a Scheimpflug rotating camera for the analysis of the anterior segment. The measurements taken by the system are fast and user-independent. Scheimpflug imaging has been reported to calculate the ACD with a mean SD of 20 µm in healthy eyes.^14^

Significant changes in ACD measurements obtained by the Pentacam rotating Scheimpflug camera have been reported following phacoemulsification cataract surgery.^15,16,17^ However, this is the first report comparing ACD changes after phacoemulsification surgery in (PEX) patients and normal patients.

Ucakhan et al.^15^ demonstrated significant deepening of the anterior chamber using a Pentacam rotating Scheimpflug camera in healthy eyes. The mean preoperative ACD was 3.0±0.8 mm and the mean postoperative ACD was 3.9±0.9 mm. Similarly, the difference in ACD measured preoperatively and postoperatively was significant in a study by Doganay et al.;^16^ who reported a mean preoperative ACD of 2.79±0.42 mm and mean postoperative ACD of 4.63±0.57 mm. The differences between the preoperative and postoperative ACD values in both of these studies were greater than those in our study. The refractive state of the patients is not mentioned by Ucakhan et al.^15^ or Doganay et al.^16^; both groups also used the Pentacam but on slightly younger patients (and therefore with potentially thinner crystalline lenses preoperatively) than in our study. Dooley et al.^17^ observed a significant increase in ACD after uneventful phacoemulsification cataract surgery in patients who had a tendency towards hypermetropia preoperatively (median preoperative SE was +0.50 D, mean preoperative ACD was 2.66±0.38 mm and mean postoperative ACD was 3.70±0.75 mm). It has been shown that hypermetropes exhibit more dramatic changes in anterior segment parameters after cataract surgery.^18^ In our study, the preoperative refractive status was -1.26 D in normal patients and -1.42 D in (PEX) patients. Mean increase in the ACD value (0.12 mm in the normal group; 0.46 mm in the (PEX) group) observed in our study was lower than those reported by previous authors.^15,16,17^

Recently developed biometric formulas (Haigis, Holladay 2) use preoperatively measured ACD to predict ELP.19,20 It has long been known how significant ELP is in calculation of IOL power formulas.^5,6,7^ Therefore, the amount of increase in the ACD postoperatively can affect the ELP and the accuracy of IOL power calculations. In this study, preoperative and postoperative ACD and MAE were evaluated and compared between (PEX) and normal groups. To our knowledge, this is the first study to evaluate the ACD and MAE following phacoemulsification surgery in eyes with (PEX). We observed that the increase in ACD values were higher in patients with (PEX) than the normal group. We thought that this difference might affect the ELP position and planned post-surgical refraction. However, the MAE calculated using different IOL calculation formulas did not differ significantly between the groups.

### Ethics

Ethics Committee Approval: KA 15-24. Informed Consent: Obtained.

Peer-review: Externally peer-reviewed.

## Figures and Tables

**Table 1:**
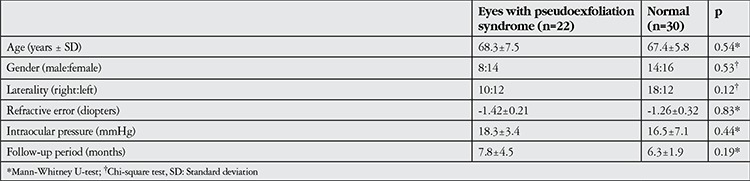
Characteristics of patients

**Table 2:**
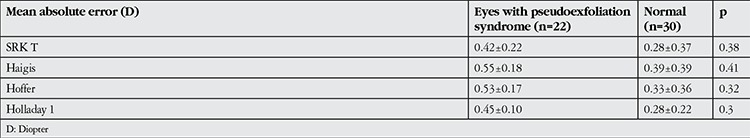
Comparison of mean absolute error with different intraocular lens power calculation formulas in pseudoexfoliative and normal patients
